# All duplicates are not equal: the difference between small-scale and genome duplication

**DOI:** 10.1186/gb-2007-8-10-r209

**Published:** 2007-10-04

**Authors:** Luke Hakes, John W Pinney, Simon C Lovell, Stephen G Oliver, David L Robertson

**Affiliations:** 1 Faculty of Life Sciences, University of Manchester, Oxford Road, Manchester M13 9PT, UK

## Abstract

**Background:**

Genes in populations are in constant flux, being gained through duplication and occasionally retained or, more frequently, lost from the genome. In this study we compare pairs of identifiable gene duplicates generated by small-scale (predominantly single-gene) duplications with those created by a large-scale gene duplication event (whole-genome duplication) in the yeast *Saccharomyces cerevisiae*.

**Results:**

We find a number of quantifiable differences between these data sets. Whole-genome duplicates tend to exhibit less profound phenotypic effects when deleted, are functionally less divergent, and are associated with a different set of functions than their small-scale duplicate counterparts. At first sight, either of these latter two features could provide a plausible mechanism by which the difference in dispensability might arise. However, we uncover no evidence suggesting that this is the case. We find that the difference in dispensability observed between the two duplicate types is limited to gene products found within protein complexes, and probably results from differences in the relative strength of the evolutionary pressures present following each type of duplication event.

**Conclusion:**

Genes, and the proteins they specify, originating from small-scale and whole-genome duplication events differ in quantifiable ways. We infer that this is not due to their association with different functional categories; rather, it is a direct result of biases in gene retention.

## Abstract 2

The comparison of pairs of gene duplications generated by small-scale duplications with those created by large-scale duplications shows that they differ in quantifiable ways. It is suggested that this is directly due to biases on the paths to gene retention rather than association with different functional categories.

## Background

The importance of gene duplication in molecular evolution is well established [1,2]. In a given genome, the collection of genes commonly referred to as 'duplicates' do not represent a homogeneous set. This is because duplicate genes can be generated through one of two main mechanisms, namely small-scale or large-scale duplication events, with the most extreme large-scale event being duplication of the entire genome. Genes resulting from these processes are thus distinct subsets of gene duplicates. However, with few exceptions [3,4], previous studies investigating the functional fate and evolution of these genes have always treated them as a single homogeneous population (for instance [5,6]).

Certain types of gene are more likely than others to be retained within the genome following a duplication event. These include the following [7-11]: genes that are present in many evolutionarily divergent lineages; those that are functionally constrained; genes involved in environmental responses; and highly expressed genes. What is not clear, however, is whether genes and their products resulting from both small-scale duplications and whole-genome duplication are subject to the same kind and degree of evolutionary pressures. Subtle differences may have consequences relating to the probabilities of different types of genes being retained after duplication.

Part of the reason for the gap in our current understanding lies with limitations in the analytical techniques commonly employed. When estimating whether two duplicates have diverged in function, we face two main challenges. First, there is a need to measure the time that has elapsed since the duplication event. In practice, this is usually done by estimating the synonymous or non-synonymous substitutions that have occurred since the duplication [12]. Second, and more important, is the need to determine whether the function(s) of the genes are different, similar, or identical. Clearly, the most accurate measure of whether two proteins share the same function can only be ascertained through concerted and careful examination of both protein members. Although this type of traditional experimentation is both appropriate and feasible for a small number of genes, it has not been performed for genome-scale data sets. With that in mind, a number of high-throughput methods (both experimental and computational) have been developed in order to investigate protein function at the whole-genome level. Such experimental approaches include yeast two-hybrid screens [13-16], genetic interaction screens [17], and the analysis of protein complexes by mass spectrometry [18-20].

Computationally, asymmetrical sequence divergence is most commonly used as a proxy for functional divergence (for example [21]). More recently, computational methods of network analysis have been used to study gene function more directly based on the annotation of their interacting partners [22], for example by identifying functional modules following network clustering [23]. Wagner [24] used network-based methodologies to define the functional fate of duplicates, taking the number of shared interactions between the products of a duplicated gene pair as a crude measure of the overlap of the two genes' functions. By clustering the interaction data, Baudot and colleagues [25] were able to derive a functional scale of convergence/divergence for a subset of the duplicated gene pairs. Conant and Wolfe [26] showed that marked asymmetry exists between the protein interaction networks associated with duplicate genes. They proposed that, following a genome duplication event, two semi-independent networks are created in which the ancestral function of the duplicated gene is split between the nascent and original copy. Most recently, Guan and colleagues [4] used protein interactions and a Bayesian data integration method to infer functional associations and showed that whole-genome duplicates had properties distinct from small-scale duplicates.

In addition to functional inference through inspection of the protein interaction network, one may also infer function directly through the annotations attached to the genes of interest, such as those presented by the Gene Ontology (GO) [27]. Comparison of the annotations contained within the 'molecular function' aspect of the ontology allows determination of the similarity of gene functions in an automated manner. A number of methods have been developed to quantify the semantic similarity (or difference) between a pair of terms [28-30]. By applying one of these methods to GO it is possible to determine the semantic similarity between the annotations of two genes, which can be considered a measure of their functional similarity.

In this study the characteristics of genes (and the proteins that they specify), derived from small-scale and whole-genome duplication (small-scale duplicates [SSDs] and whole-genome duplicates [WGDs], respectively), are compared for the yeast *Saccharomyces cerevisiae*. Comparison of the functional divergence between the paralogous pairs of duplicates, using both protein interactions and GO annotations as proxies for protein function, reveals a distinct difference between the functional divergence of duplicate genes of each duplicate type. We then show that despite the SSD and WGD sets being associated with different functional categories, there is no evidence that these differences influence essentiality. Rather, proteins derived from whole-genome duplication in complexes are significantly more dispensable than those derived from small-scale duplication. We infer that the difference between the duplicate sets is most probably a result of the different strengths of constraint imposed by dosage and balance effects on the gene products, that is they are a direct consequence of biases in gene retention.

## Results

### WGD paralog pairs are functionally more similar than SSD paralogs

**Figure 1:**
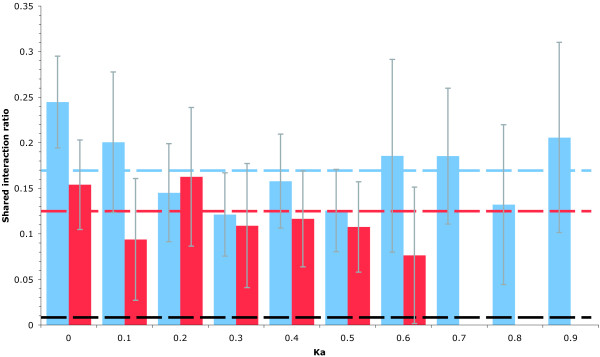
Comparison of the shared interaction ratio for duplicate gene products and random protein pairs. Whole-genome duplicates (WGDs) are illustrated in blue and small-scale duplicates (SSDs) are illustrated in red. Mean shared interaction ratio *r *is plotted against gene sequence divergence measured by non-synonymous substitution rate (K_a_). The dashed lines indicate the average shared interaction ratio for WGDs (blue), SSDs (red), and pairs of proteins selected at random from the genome (black). Error bars show standard errors on the mean of *r *for each bin.

By using the protein interaction network as a proxy for protein function, it is possible to investigate the functional similarity of each member of a duplicate gene pair on a large scale. At the point of duplication, paralogous pairs have identical protein sequences and hence identical binding surfaces, specificity, and (ultimately) function. This functional similarity should be reflected within the protein interaction network as a tendency for duplicate gene pair products to share more protein interactions than random pairings of non-duplicates. Figure 1 shows the average number of shared interactions for both the SSD and WGD sets of proteins, plotted against sequence divergence measured by non-synonymous substitutions, K_a_. Dashed lines on the graph represent the average shared interaction ratio for each duplicate set and for a set of randomly paired proteins. It is evident from the disparity between the averages for each group of pairs that proteins derived from both small-scale and whole-genome duplication, share many more interactions than we would expect by chance (*P *< 2 × 10^-16^, Wilcoxon rank sum). It is also clear that proteins derived from the whole-genome duplication on average have more protein interactions in common, and hence more similar functions, than do those from small-scale duplications (*P *= 1 × 10^-4^, Wilcoxon rank sum). Note that this difference between WGDs and SSDs is not due to some bias introduced by a stringent sequence identity threshold because these results remain unchanged if a less conservative threshold is used to identify SSD pairs (Additional data file 1).

It is a possibility that this difference in connectivity might be due to differences in the average connectivity of the gene products contained within each group. Given the high error rate and degree of noise within the existing protein interaction network data [31], pairs of highly connected proteins could, simply by chance, be more likely to share protein interactions than pairs whose members are involved in fewer interactions. To test this, the average degree of the proteins within each duplicate set and within similar sized random genome samples was investigated. No significant differences were found between the average degrees of the proteins in any class (SSDs, WGDs, or random pairings), with all three sets having gene products with an average of about ten interactions. This finding indicates that, in general, duplicates are not more connected than non-duplicates, and confirms the observation that pairs of WGDs share more protein interactions than pairs of SSDs.

**Figure 2:**
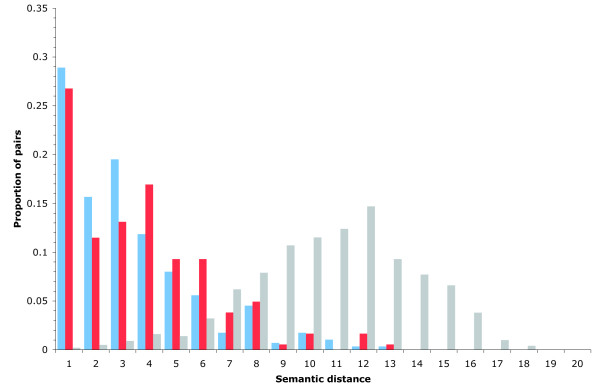
Relationship between semantic distance and the proportion of pairs within each duplicate set. Whole-genome duplicates (WGDs) are illustrated in blue, small-scale duplicates (SSDs) in red, and random gene pairings in gray. A higher semantic distance indicates greater functional divergence.

In addition to protein-protein interactions, functional annotations within the GO database [32] were used as a second computationally amenable proxy for protein function. The semantic distance between the annotations of a pair of duplicated genes [28,33] was used to quantify the similarity of their molecular functions. By studying the distributions of semantic distances for each class of duplicate, their propensity to share functional annotations was compared (Figure 2). In agreement with the result obtained using the protein interaction network, on average the members of WGD pairs were found to have a lower semantic distance, and hence a more similar function, than the members of SSD pairs (mean semantic distance: 3.21 for SSDs versus 2.76 for WGDs; *P *= 0.045, Wilcoxon rank sum). Note that both sets of duplicate genes tended to have much lower semantic distances than pairs selected at random, again indicating that duplicated genes have functions that are more similar than would be expected by chance (mean semantic distance: 10.26; P < 2 × 10^-6^, Wilcoxon rank sum). These results also remain unchanged if a less conservative sequence identity threshold is used to identify SSD pairs (Additional data file 2).

### WGDs are less likely to be essential than SSDs

Genes with overlapping functions are more likely to have the ability to compensate for each other when mutation/loss occurs. Because WGDs have tendencies both to share more interactions and to be functionally more related (Figures 1 and 2), WGDs should be more dispensable than SSDs. To investigate this hypothesis, the different duplicate sets were analyzed within the context of gene knockout studies; deletion of a WGD gene should, on average, have a weaker phenotypic effect than deletion of a SSD gene. Using the data generated in the *Saccharomyces *Gene Deletion Project [34], those genes that showed an essential phenotype upon deletion were identified. In accordance with previous observations [35], deletion of a duplicate was found to be significantly less likely to confer an essential phenotype than deletion of a non-duplicate (only about 8% of duplicates are essential versus about 29% of non-duplicates; P < 1 × 10^-3^, Pearson's χ^2^). Moreover, the proportion of essential genes within the WGD set was found to be less than that observed for SSDs (6% of WGD genes are essential versus about 9% of SSD genes; *P *< 1 × 10^-3^, Pearson's χ^2^). Thus, WGDs play a relatively greater role in redundancy (and hence 'robustness') than do SSDs, as has been inferred from a comparison of duplicates and single-copy genes [35].

### WGDs and SSDs are linked with different functional categories

**Figure 3:**
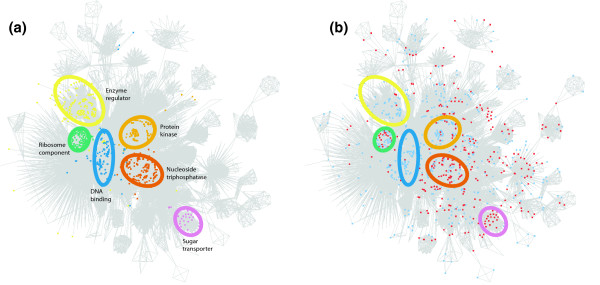
Visualization of the two sets of duplicates on a semantic distance network. **(a) **The yeast proteome is distributed spatially according to semantic distance, with six high-level functional classes highlighted in different colors that are either over-represented or under-represented in the whole-genome duplicate (WGD) or small-scale duplicate (SSD) sets (see Table 1). **(b) **WGDs are shown in blue and SSDs in red; the same six functional classes are highlighted. The products of the two types of duplicate gene have a tendency to occupy separate areas of semantic space, indicating involvement in different functions.

An explanation for the difference in dispensability between SSDs and WGDs could be that the two sets are associated with different functional classes of proteins. To test this hypothesis, the GO was used to investigate over-represented and under-represented functional annotations [32] for the genes within each duplicate class. We find that, in terms of their functions, the two types of duplicate show distinct profiles compared both to the set of all yeast open reading frames (ORFs; Table 1) and to each other. There is little overlap between the functions of genes that are significantly over-represented or under-represented in the sets of SSDs and WGDs. Proteins derived from small-scale duplication are enriched for transporter functions, particularly sugar transporters, and also for those with hydrolase and helicase activities. Genes specifying proteins that are involved in binding, particularly nucleic acid binding and transcription regulators, are under-represented in this set of duplicates. Whole-genome duplication derived proteins that are structural molecules or protein kinases are significantly over-represented, whereas methyltransferases are under-represented. Figure 3 shows a visualization of representative molecular functions associated with the two sets of duplicate genes on a semantic distance network. Clearly, the distributions of the duplicate genes are not random across all functional categories.

**Table 1:** Over-represented and under-represented functional annotations within the different duplicate sets

GO ID	Description	Total	observed	*P *(raw)	*P *(corrected)
Over-represented in set of WGDs					
0004672	Protein kinase activity	127	52	2.7 × e^-12^	<0.001
0003735	Structural constituent of ribosome	217	72	3.4 × e^-11^	<0.001
0016773	Phosphotransferase activity, alcohol group as acceptor	171	61	3.9 × e^-11^	<0.001
0016301	Kinase activity	197	67	4.9 × e^-11^	<0.001
0004674	Protein serine/threonine kinase activity	69	32	1.1 × e^-09^	<0.001
0016772	Transferase activity, transferring phosphorus-containing groups	294	78	4.3 × e^-07^	<0.001
0016538	Cyclin-dependent protein kinase regulator activity	23	14	8.8 × e^-07^	<0.001
0005198	Structural molecule activity	338	83	5.6 × e^-06^	0.001
0030234	^E^nzyme regulator activity	180	50	1.4 × e^-05^	0.002
0019887	Protein kinase regulator activity	44	18	4.3 × e^-05^	0.004
0016740	Transferase activity	641	135	4.6 × e^-05^	0.004
0005083	Small GTPase regulator activity	47	18	1.2 × e^-04^	0.018
0019207	Kinase regulator activity	47	18	1.2 × e^-04^	0.018
0035251	UDP-glucosyltransferase activity	13	8	2.0 × e^-04^	0.027
0003704	Specific RNA polymerase II transcription factor activity	45	17	2.2 × e^-04^	0.029
0016791	Phosphoric monoester hydrolase activity	88	27	2.4 × e^-04^	0.029
0030508	Thiol-disulfide exchange intermediate activity	8	6	2.9 × e^-04^	0.042
Under-represented in set of WGDs					
0008757	S-adenosylmethionine-dependent methyltransferase activity	62	0	2.7 × e^-05^	<0.001
0016741	Transferase activity, transferring one-carbon groups	84	2	8.7 × e^-05^	0.003
0015078	Hydrogen ion transporter activity	54	0	1.1 × e^-04^	0.006
0008168	Methyltransferase activity	82	2	1.2 × e^-04^	0.006
0031202	RNA splicing factor activity, transesterification mechanism	51	0	1.8 × e^-04^	0.008
0016251	General RNA polymerase II transcription factor activity	62	1	3.4 × e^-04^	0.014
Over-represented in set of SSDs					
0051119	Sugar transporter activity	25	22	2.7 × e^-20^	<0.001
0015144	Carbohydrate transporter activity	30	23	1.5 × e^-18^	<0.001
0015145	Monosaccharide transporter activity	21	18	2.3 × e^-16^	<0.001
0015149	Hexose transporter activity	21	18	2.3 × e^-16^	<0.001
0015578	Mannose transporter activity	15	15	3.0 × e^-16^	<0.001
0005353	Fructose transporter activity	15	15	3.0 × e^-16^	<0.001
0017111	Nucleoside-triphosphatase activity	243	65	7.3 × e^-16^	<0.001
0005355	Glucose transporter activity	18	16	3.5 × e^-15^	<0.001
0016818	Hydrolase activity, acting on acid anhydrides, in phosphorus-containing anhydrides	264	67	4.4 × e^-15^	<0.001
0016462	Pyrophosphatase activity	264	67	4.4 × e^-15^	<0.001
0016817	Hydrolase activity, acting on acid anhydrides	264	67	4.4 × e^-15^	<0.001
0005215	Transporter activity	410	84	6.7 × e^-13^	<0.001
0003824	Catalytic activity	1885	252	7.3 × e^-13^	<0.001
0016887	ATPase activity	185	46	2.5 × e^-10^	<0.001
0016787	Hydrolase activity	707	109	2.1 × e^-08^	<0.001
0016614	Oxidoreductase activity, acting on CH-OH group of donors	75	24	3.2 × e^-08^	<0.001
0016616	Oxidoreductase activity, acting on the CH-OH group of donors, NAD or NADP as acceptor	67	22	7.2 × e^-08^	<0.001
0004386	Helicase activity	83	24	2.8 × e^-07^	<0.001
0042626	ATPase activity, coupled to transmembrane movement of substances	58	19	5.9 × e^-07^	<0.001
0043492	ATPase activity, coupled to movement of substances	58	19	5.9 × e^-07^	<0.001
0016820	Hydrolase activity, acting on acid anhydrides, catalyzing transmembrane movement of substances	58	19	5.9 × e^-07^	<0.001
0016491	Oxidoreductase activity	262	49	1.2 × e^-06^	<0.001
0015075	Ion transporter activity	145	32	2.6 × e^-06^	<0.001
0008324	Cation transporter activity	124	28	6.9 × e^-06^	<0.001
0042623	ATPase activity, coupled	125	28	8.2 × e^-06^	0.001
0018456	Aryl-alcohol dehydrogenase activity	8	6	1.5 × e^-05^	0.002
0015294	Solute:cation symporter activity	8	6	1.5 × e^-05^	0.002
0003924	GTPase activity	54	16	2.0 × e^-05^	0.002
0005354	Galactose transporter activity	6	5	3.9 × e^-05^	0.009
0015293	Symporter activity	9	6	4.3 × e^-05^	0.012
0005537	Mannose binding	4	4	7.6 × e-05	0.017
0015238	Drug transporter activity	15	7	2.0 × e^-04^	0.035
0003678	DNA helicase activity	35	11	2.2 × e^-04^	0.039
Under-represented in set of SSD					
0003676	Nucleic acid binding	494	12	1.8 × e^-10^	0
0005488	Binding	1034	58	1.1 × e^-06^	0
0003723	RNA binding	231	4	1.6 × e^-06^	0
0003677	DNA binding	220	6	8.0 × e^-05^	0.002
0030528	Transcription regulator activity	326	14	3.3 × e^-04^	0.006
0016779	Nucleotidyltransferase activity	80	0	3.7 × e^-04^	0.009

GO, Gene Ontology; SSD, small-scale duplicate; WGD, whole-genome duplicate.

### Differences in essentiality between WGDs and SSDs are not due to differences in their functional categories

Mapping the yeast essential genes onto functional categories, we find no pattern of correlation between the functions that are over-represented or under-represented in the SSD and WGD sets and the distribution of essential genes in those classes (Table 2). For the functional classes that are significantly over-represented in the set of essential ORFs (which we might also expect to be significantly over-represented in the SSDs), we observe little difference between the SSD and WGD sets. Although genes derived from small-scale duplication appear to be enriched for some essential functions, this enrichment is counterbalanced by an equally strong suppression of others. For the functions that tend to be mostly associated with non-essential ORFs, we actually observe the opposite of what might be expected if differences in protein function were responsible for the discrepancy (an over-representation of these classes among SSD genes). Thus, the phenotypic asymmetry between the two classes of duplicate is not because they encode proteins that have functions that are either more or less likely to be essential upon deletion. The difference must therefore stem from some other factor.

**Table 2:** The relationship between dispensability and functional category for both WGDs and SSDs

GO ID	Description	% all ORFs	% SSDs	% WGDs
Over-represented in set of essential genes				
0003824	Catalytic activity	32.5	46.5^+^	35.8
0005488	Binding	17.8	10.7^-^	17.9
0016740	Transferase activity	11.1	9.4	15.0^+^
0003676	Nucleic acid binding	8.5	2.2^-^	10.1
0005515	Protein binding	7.5	5.5	5.9
0005198	Structural molecule activity	5.8	8.1	9.2^+^
0030528	Transcription regulator activity	5.6	2.6^-^	7.0
0016772	Transferase activity, transferring phosphorus-containing groups	5.1	4.6	8.7^+^
0016462	Pyrophosphatase activity	4.6	12.4^+^	3.1
0016817	Hydrolase activity, acting on acid anhydrides	4.6	12.4^+^	3.1
0016818	Hydrolase activity, acting on acid anhydrides, in phosphorus-containing anhydrides	4.6	12.4^+^	3.1
0017111	Nucleoside-triphosphatase activity	4.2	12.0^+^	2.7
0003723	RNA binding	4.0	0.7^-^	3.1
0016887	ATPase activity	3.2	8.5^+^	1.8
0016874	Ligase activity	2.2	1.7	2.0
0003702	RNA polymerase II transcription factor activity	2.1	0.7	2.7
0004386	Helicase activity	1.4	4.4^+^	0.4
0016779	Nucleotidyltransferase activity	1.4	0.0^-^	1.0
0016251	General RNA polymerase II transcription factor activity	1.1	0.4	0.1^-^
Under-represented in set of essential genes				
0005215	Transporter activity	7.1	15.5^+^	6.2
0016491	Oxidoreductase activity	4.5	9.0^+^	5.3
0015075	Ion transporter activity	2.5	5.9^+^	1.6
0008324	Cation transporter activity	2.1	5.2^+^	1.1

Gene Ontology (GO) categories significantly over-represented and under-represented (corrected *P *< 0.05) are sorted by abundance (1% cut-off). Significant over-representation and under-representation in the duplicate sets are denoted by superscript '+' and '-', respectively. ORF, open reading frame; SSD, small-scale duplicate; WGD, whole-genome duplicate.

### WGDs are more likely to be members of protein complexes than SSDs; WGD associated complexes are less likely to be essential than SSD complexes

If the functions that the small-scale and whole-genome duplication derived sets of proteins are associated with do not account for their differences, then we surmise that an important factor must be related to their different mechanisms of generation (sequential versus simultaneous, respectively). Because of dosage and balance effects [36,37], the two duplicate types will be subject to differential probabilities of being retained subsequent to their generation by duplication. These factors will have the greatest impact on duplicates present in complexes. We investigated the relative dispensabilities of both complex-forming and non-complex-forming WGD and SSD associated proteins (Table 3). For gene products participating in complexes (as described in MIPS [Munich Information Center for Protein Sequences] [38]), we find a statistically significant asymmetry between the dispensability of the two duplicate types, with 10% of WGDs versus 21% of SSDs being essential. For non-complex-forming genes, the two classes of duplicate appear to be similarly dispensable, with 6% of WGDs versus 9% of SSDs being essential (Table 3). Interestingly, the products of whole-genome duplication are significantly more likely to be present in a protein complex than those of small-scale duplications (19% versus 14%; χ^2 ^= 4.44, *P *< 0.05).

**Table 3:** Dispensability of SSD and WGD proteins found in complexes and those not found within protein complexes

	WGD	SSD
Complexes		
Essential	16 (10%)	15 (21%)
Not essential	138 (90%)	55 (79%)
Total	154	70
Non-complexes		
Essential	32 (5%)	28 (7%)
Not essential	642 (95%)	398 (93%)
Total	674	426

SSD, small-scale duplicate; WGD, whole-genome duplicate.

### Differing proportions of complex-forming proteins explain differences in functional similarity between WGD and SSD paralog pairs, but not their differences in essentiality

**Figure 4:**
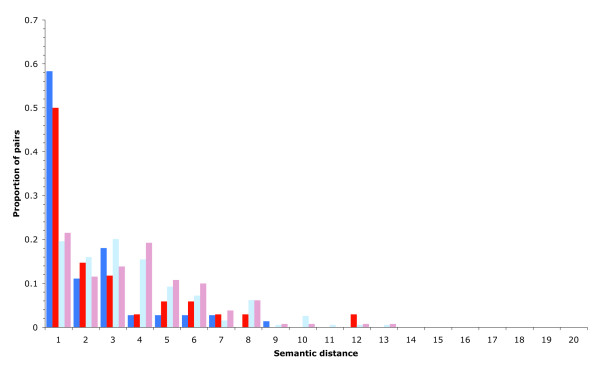
Relationship between semantic distance, duplicate set and complex membership. The proportion of duplicate pairs having a certain level of functional divergence as measured by semantic distance for the following: pairs of complex-forming whole-genome duplicate (WGD; dark blue), complex-forming small-scale duplicate (SSD; red), non-complex-forming WGD (light blue), and non-complex-forming SSD (pink) proteins. Significant differences in the degree of functional divergence between the pairs in the two categories (complex and non-complex) are observed. No significant difference between the semantic distances of pairs of SSDs found in complexes and complex-forming WGD pairs is observed; nor, indeed, is there any difference between SSD pairs not in complexes and WGD pairs not found within complexes.

To investigate how the difference in propensity for complex membership maps onto the asymmetry in dispensability between the two duplicate types, we repeated the semantic distance analysis with these subsets (Figure 4). This analysis revealed significant differences between the degrees of functional divergence between the pairs of gene products in the two categories (complex and non-complex), suggesting that the functional evolution of proteins that participate in protein complexes is considerably more constrained than those that do not. Importantly, we found no significant difference between the semantic distances of pairs of SSD associated proteins found in complexes and complex-forming WGD protein pairs, nor indeed between SSD pairs not in complexes and WGD pairs not found within complexes. This indicates that although the observed difference in functional divergence of SSDs and WGDs (Figure 2) is accounted for by the greater number of WGDs that encode complex-forming proteins, functional constraint caused by complex membership is not a factor in determining gene dispensability, because complex-forming WGDs are still less dispensable than complex-forming SSDs, even when they exhibit similar levels of functional divergence.

## Discussion

Collectively, our results demonstrate that the differences between the two types of duplicate are not limited to the way in which they were generated. Investigation of the functional similarity between the members of duplicate pairs reveals a distinct difference between the two duplicate types, with whole-genome duplication derived genes tending to be more functionally similar than those from small-scale duplication. This result is the same regardless of whether function is measured using shared interactions, in the context of protein interaction data (Figure 1), or by calculation of the semantic distance between the functional annotations of members of a duplicate pair (Figure 2). Although our results were obtained using different methodology (semantic distance rather than Bayesian inference), this finding is consistent with the recent report by Guan and colleagues [4].

The greater functional similarity among WGDs suggests that they contribute more to redundancy than SSDs. Indeed, investigating essentiality directly, in the context of gene knockout studies (Table 2), we find that genes derived from whole-genome duplication are more likely to be dispensable than those from small-scale duplications (Table 3). Our results indicate that this asymmetry does not result from a bias toward more dispensable functions within whole-genome duplication derived genes, suggesting that it has a more fundamental basis. The difference in functional divergence between duplicates observed between the two sets (Figures 1 and 2) can be accounted for by their products having greater propensity to be part of protein complexes, which are generally less divergent than proteins that are not part of complexes. However, although we find that proteins associated with SSDs and WGDs in complexes are equally functionally constrained (Figure 4), they still exhibit a twofold difference in their propensity to confer an essential phenotype upon deletion. This indicates that, contrary to expectations, neither differences in functional divergence nor the propensity for complex membership can explain the observed asymmetry in duplicate dispensability. Rather, that difference is likely to stem from the relative strengths of evolutionary constraint prevalent in the period following each type of duplication event.

Consider a protein complex composed of three subunits A, B, and C. In some cases an excess of any of the members of such a complex can be detrimental [36]. Such cases include (but are not limited to) situations in which individual subunits can homodimerize to form complexes with different functions to that of ABC [39] or cases in which subunits that form a bridge between parts of the complex may, when in excess, inhibit complex assembly altogether [40]. Following whole-genome duplication, all three subunits of the complex will be present in duplicate and thus their stoichiometries will be maintained in a 'balanced' fashion, causing minimal phenotypic disruption. Conversely, small-scale duplication events are likely to involve only one member of a complex and thus, because they will cause disruption to the 'balance' of any complex in which they are involved, they will have a greater tendency to be immediately deleterious to the organism. In this way, duplication derived proteins involved in multi-subunit complexes will have a greater probability of persisting (being retained) in the genome following whole-genome duplication but are more likely to be selected against and are more rapidly removed following small-scale duplication events. The significance of such balance effects, specifically within whole-genome duplication, was highlighted by Papp and colleagues [37]. Those investigators demonstrated that the frequency of genes encoding the subunits of cytosolic ribosomes is tenfold higher among WGDs than among SSDs [37].

Although balance (or rather imbalance) effects have been shown to be important for a few select entities within the cell (for example, components of the cytoskeleton), in general their prevalence is thought to be low [41]. Another explanation for the reduction in retention of complex components following single-gene duplication is that, rather than being detrimental, duplication of an individual complex component is more likely to be neutral. Because the small-scale duplication provides no immediate benefit, it will not be selected for and so will probably be lost relatively rapidly. In contrast, duplication of an entire complex during whole-genome duplication is likely to have immediate benefit for those complexes that are dosage sensitive, and so selection will act strongly on its members to retain them. This type of dosage effect and biased retention has been reported in an analysis of whole-genome duplication in the ciliate *Paramecium tetraurelia *[42].

How, then, does this proposed mechanism of retention relate to the differences observed in the functional similarity and dispensability of each duplicate type? In the period that follows duplication, duplicated genes may be retained for one of three reasons. The first is that, in the case of a dosage advantage, duplicates will be subject to selection and will maintain the function of the ancestral gene. Alternatively, when dosage is not advantageous, they may diverge and either (second reason) gain a new function or (third reason) assume part of the ancestral gene's function. Because whole-genome duplication generates two copies of every gene within the genome, and thus of every member of every protein complex, it enables entire complexes to be duplicated, which will result in a greater propensity for WGDs to be retained in cases where increased dosage is an advantage. This leads to the over-representation of genes encoding members of protein complexes within the WGD set. Conversely, individual complex members duplicated by small-scale duplication will probably provide no immediate benefit (or be selected against according to the balance hypothesis). Either way, they will have a relatively low probability of being retained following duplication.

The underlying factor that results in whole-genome duplication derived genes being more dispensable than small-scale duplication derived genes does not appear to be related to the particular functional categories of genes that are retained following each duplication event (Table 2). That this asymmetry is observed in proteins involved in complexes indicates that this phenomenon is, instead, probably due to the differences in the probability of retention of each duplicate type. For example, following whole-genome duplication, a complex retained for dosage reasons is inherently 'backed up', whereas complexes involving small-scale duplication derived genes are likely to have functions that are novel, or even unique, and are thus less dispensable. As a result, genome duplicates will contribute relatively more to redundancy, although merely as a by-product of their paths to retention.

## Conclusion

We have demonstrated that genes originating from single-gene and whole-genome duplication events differ in quantifiable ways; whole-genome and small-scale duplication derived proteins are enriched for different categories of molecular functions. WGD paralogs are functionally less diverse, less likely to be essential, and more likely to be members of a protein complex than SSD paralogs. Protein complex members originating from a whole-genome duplication event are also about half as likely to be essential as those produced by small-scale duplication events.

Given that rates of small-scale gene duplication have been estimated to be as high as about 0.01 per gene per million years [43], there is clearly a huge difference in the probability of gene retention following a small-scale duplication event (average half-life about 4 million years [43]) as compared with a whole-genome duplication event (average half-life about 33 million years, based on 12% paralog retention in *S. cerevisiae *[21] after about 100 million years [44]). This discrepancy provides compelling evidence that these different types of duplicates must experience different evolutionary pressures en route to retention, which are observable as differences in functional diversity, essentiality, and protein complex membership.

Such differences have important implications for how new genes with novel protein functions arise within the genome. They indicate that there is bias in the types of genes that contribute the most to functional innovation and evolution of complexity. As a direct result of their greater chance of being retained, WGDs will often be observed to contribute to functional innovation. Paradoxically, the same processes (balance and dosage) that increase the probability of retention of genome duplicates also impose constraints on their functional evolution. Although more frequently lost from the genome, the products of small-scale duplications will, when they are retained, have the potential to make a relatively larger contribution to innovation. Our finding that the different duplicate gene sets have a tendency to be involved in different functional categories (Figure 3) implies that, despite their differences, both WGDs and SSDs contribute significantly to evolutionary 'raw material'.

## Materials and methods

### Duplicate genes

The 450 pairs of WGD genes were taken from the previous study conducted by Kellis and co-workers [21]. SSD genes were identified using GenomeHistory [45] with the following parameters: BLAST (basic local alignment search tool) threshold 1 × 10^-8^, minimum ORF translation length 100, minimum aligned residues 100, and percentage identity threshold 40%. All WGD genes, dubious ORFs, and transposable elements were excluded from the SSD data set. In cases in which a gene was found to have more than one paralog, a single representative paralog was selected at random. This yielded a set of WGD genes (450 pairs) and a conservative set of SSD genes (271 pairs). Sequence divergence (K_a_) for all duplicate pairs was calculated using the method proposed by Yang and Nielsen [46], as implemented in PAML (phylogenetic analysis by maximum likelihood) [47].

A stringent identity threshold of 40% was selected to ensure that the SSD pairs identified were genuine paralogs. To ensure that the exclusion of more distant paralog pairs was not causing bias in our conclusions, we also compiled sets of SSD pairs at 30% (422 pairs) and 20% (724 pairs) sequence identity. The distributions of sequence divergence (K_a_) for the WGD pairs and three sets of SSD pairs can be seen in Additional data file 3. Note that both the 30% and 20% SSD sets contain substantial numbers of highly divergent pairs, indicating the increased presence of potentially false-positive paralogy assignments in these less conservative data sets.

### Protein interaction data

Protein interaction data were extracted from the BioGRID database [48], and all non-physical interactions were excluded. Non-physical interactions were defined as those where the method of detection was annotated as one of the following: synthetic lethality, dosage rescue, synthetic growth defect, synthetic rescue, epistatic miniarray profile, dosage lethality, phenotypic enhancement, phenotypic suppression, or dosage growth defect. For duplicate pairs in which both members were identified as interacting with at least one other protein (377 pairs), the shared interaction ratio was then calculated using the following equation:

$$r=\frac{2s}{n_{1}+n_{2}}$$

Where *r *is the shared interaction ratio, *s *is the number of interactions shared between the two proteins, and *n*_1 _and *n*_2 _are the number of interactions for ORF1 and ORF2, respectively.

### Semantic distance

To assess the functional differences between each member of a duplicate pair, the GO annotations [32] of each of the genes were compared using a semantic distance measure [28] limited to the 'molecular function' aspect of the GO. The semantic distance *d*(*t*_1_, *t*_2_) between two terms *t*_1 _and *t*_2 _within the ontology is given by the following:

$$d(t_{1},t_{2})=2ln\left(\underset{t\in S(t_{1},t_{2})}{\min}\{p(t)\}\right)-lnp(t_{1})-lnp(t_{2})$$

where *p*(*t*) is the information content of a term *t *(the fraction of all genes associated with that term) and *S*(*t*_1_, *t*_2_) is the set of all parent terms shared by *t*_1 _and *t*_2_. For two genes *a *and *b *with sets of annotated terms *A *and *B*, we define the semantic distance *D*(*a*, *b*) between those two genes as follows:

$$D(a,b)=\frac{1}{2}\left(\frac{\sum_{t_{a}\in A}\underset{t_{b}\in B}{\min}\{d(t_{a},t_{b})\}}{\left|A\right|}+\frac{\sum_{t_{b}\in B}\underset{t_{a}\in A}{\min}\{d(t_{b},t_{a})\}}{\left|B\right|}\right)$$

Where |*A*| and |*B*| are the numbers of annotated terms in the sets *A *and *B*, respectively.

The semantic distance was chosen over other possible methods because (unlike, for instance, semantic similarity, as defined by Resnik [30]) it provides us with a defined reference point (at *D *= 0) immediately following a gene duplication, away from which a duplicate pair may be expected to evolve. In order to make this study independent of the protein interaction data described above, all annotations that were tagged as IPI (inferred from protein interaction) were eliminated from the data set. For the semantic distance network (Figure 3), the IPI annotations were included but genes with unknown function were excluded. An edge is drawn between two genes if they are functionally similar, defined as being within a cut-off distance of 5.0. The network was visualized using LGL [49]. Only the largest connected component is shown.

### Phenotypic effect of duplicate deletion

A list of essential genes was obtained from the *Saccharomyces *Gene Deletion Project [34]. For calculations relating to the number of essential genes within different sample gene sets, we excluded all dubious ORFs and all ORFs that were not available within the deletion collection.

### Gene Ontology analysis

Lists of over-represented and under-represented GO terms were obtained for the WGD and SSD sets, and for essential genes. The hypergeometric distribution was used to calculate raw *P *values for the number of genes associated with each GO term within each data set, considered as a sample from all genes in the genome. Each raw *P *value, *p*_*raw*_, was corrected for multiple testing by taking 1,000 random samples of the same size from the whole genome and recording the proportion of samples in which any GO term received a *P *value lower than *p*_*raw*_. This Monte Carlo approach is considered to be more accurate than other methods for correcting for multiple testing, owing to the fact that GO terms are not independent of each other [50].

## Abbreviations

GO, Gene Ontology; IPI, inferred from protein interaction; K_a_, non-synonymous substitution rate; K_s_, synonymous substitution rate; ORF, open reading frame; SSD, small-scale duplicate; WGD, whole-genome duplicate.

## Authors' contributions

LH, SGO and DLR conceived research. LH, JWP, SCL and DLR designed research. LH and JWP performed research. LH, JWP, SCL and DLR discussed results. LH wrote the manuscript with contributions from all authors.

## Additional data files

The following additional data are available with the online version of this paper. Additional data file 1 shows a comparison of the shared interaction ratio for duplicates and random ORF pairs. Additional data file 2 shows the semantic distance distributions for each duplicate set. Additional data file 3 shows the numbers of paralog pairs identified at different levels of sequence divergence (K_a_) within each duplicate set.

## Supplementary Material

Additional data file 1 WGDs are illustrated in blue and SSDs are illustrated in red (found at 40% sequence identity), yellow (30% ID), and green (20% ID). Mean shared interaction ratio *r *is plotted against sequence divergence measured by K_a_. The rightmost bin indicates the mean shared interaction ratio for WGDs, three sets of SSDs and pairs of proteins selected at random from the genome (black). Error bars show standard errors on the mean of *r *for each bin. Click here for file

Additional data file 2 WGDs are illustrated in blue, SSDs in red (found at 40% sequence identity), yellow (30% ID) and green (20% ID), and random gene pairings in gray. A higher semantic distance indicates greater functional divergence. Click here for file

Additional data file 3 WGDs are illustrated in blue and SSDs are illustrated in red (found at 40% sequence identity), yellow (30% ID) and green (20% ID). Click here for file
