# Supplementary figures and images for: All duplicates are not equal: the difference between small-scale and genome duplication
Source: Genome Biol. 2007 Oct 4;8(10):R209. doi: 10.1186/gb-2007-8-10-r209 (PMC2246283; doi:10.1186/gb-2007-8-10-r209)

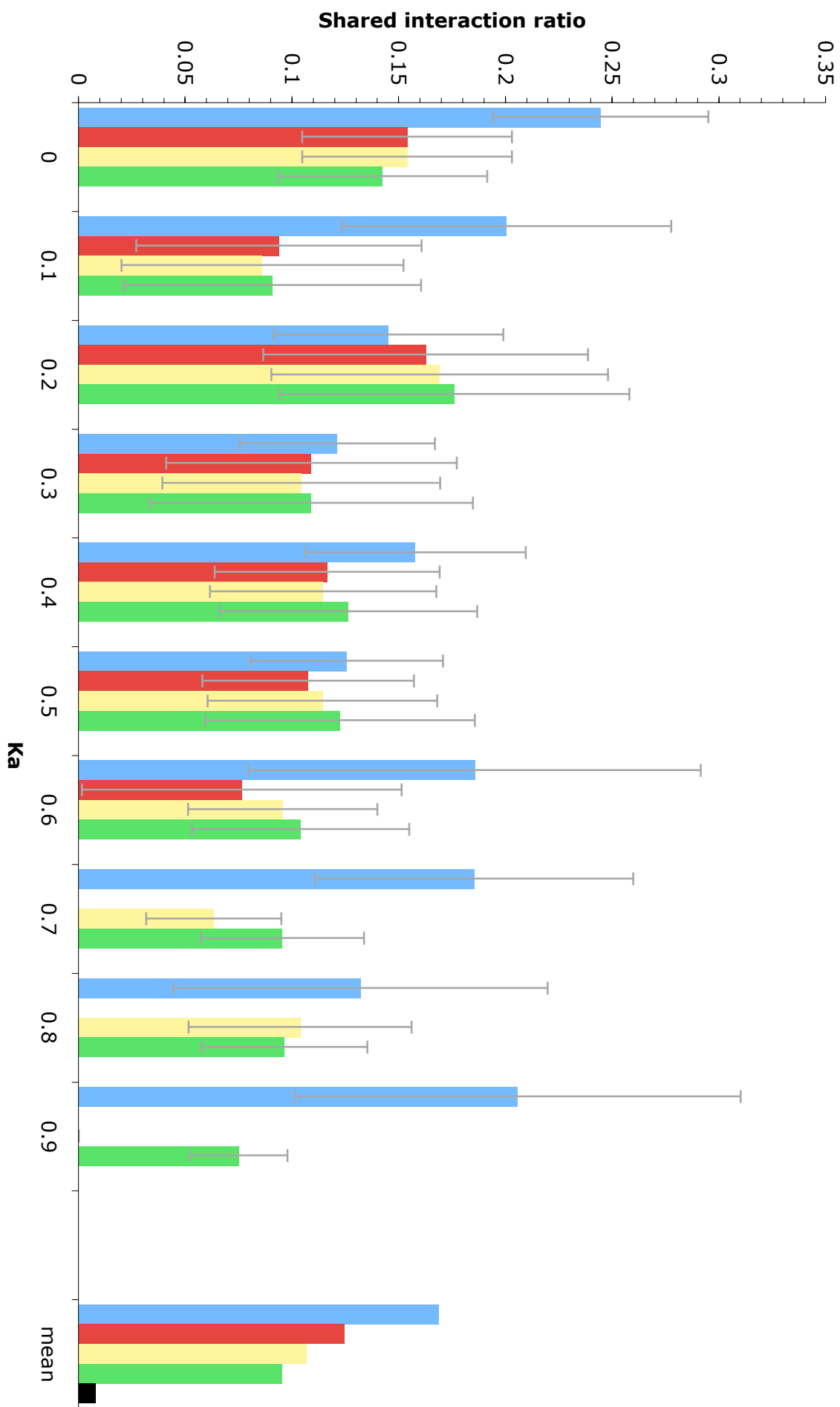

Supplement: Additional data file 1 — WGDs are illustrated in blue and SSDs are illustrated in red (found at 40% sequence identity), yellow (30% ID), and green (20% ID). Mean shared interaction ratio r is plotted against sequence divergence measured by Ka. The rightmost bin indicates the mean shared interaction ratio for WGDs, three sets of SSDs and pairs of proteins selected at random from the genome (black). Error bars show standard errors on the mean of r for each bin. [file gb-2007-8-10-r209-S1.pdf]

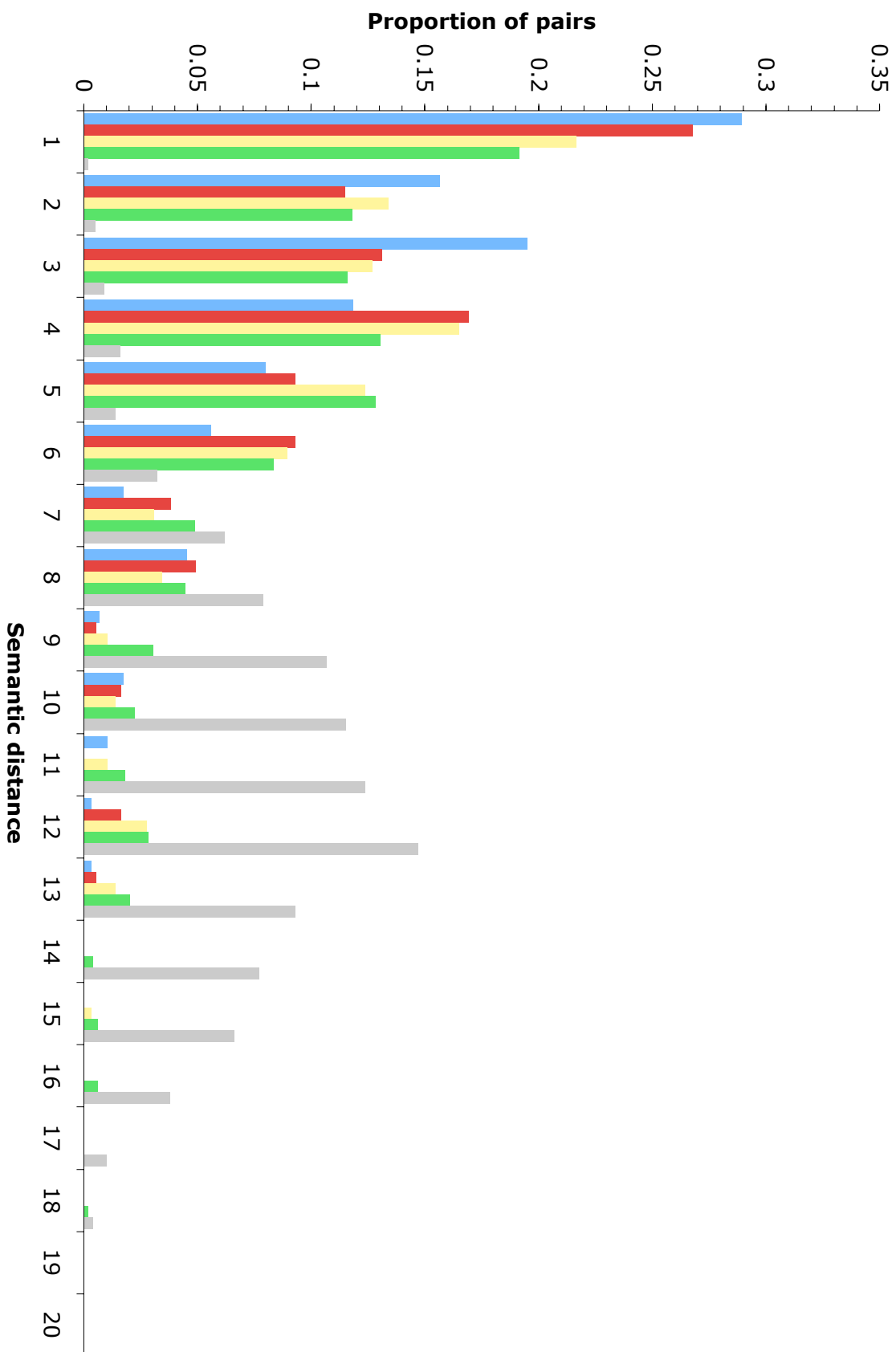

Supplement: Additional data file 2 — WGDs are illustrated in blue, SSDs in red (found at 40% sequence identity), yellow (30% ID) and green (20% ID), and random gene pairings in gray. A higher semantic distance indicates greater functional divergence. [file gb-2007-8-10-r209-S2.pdf]

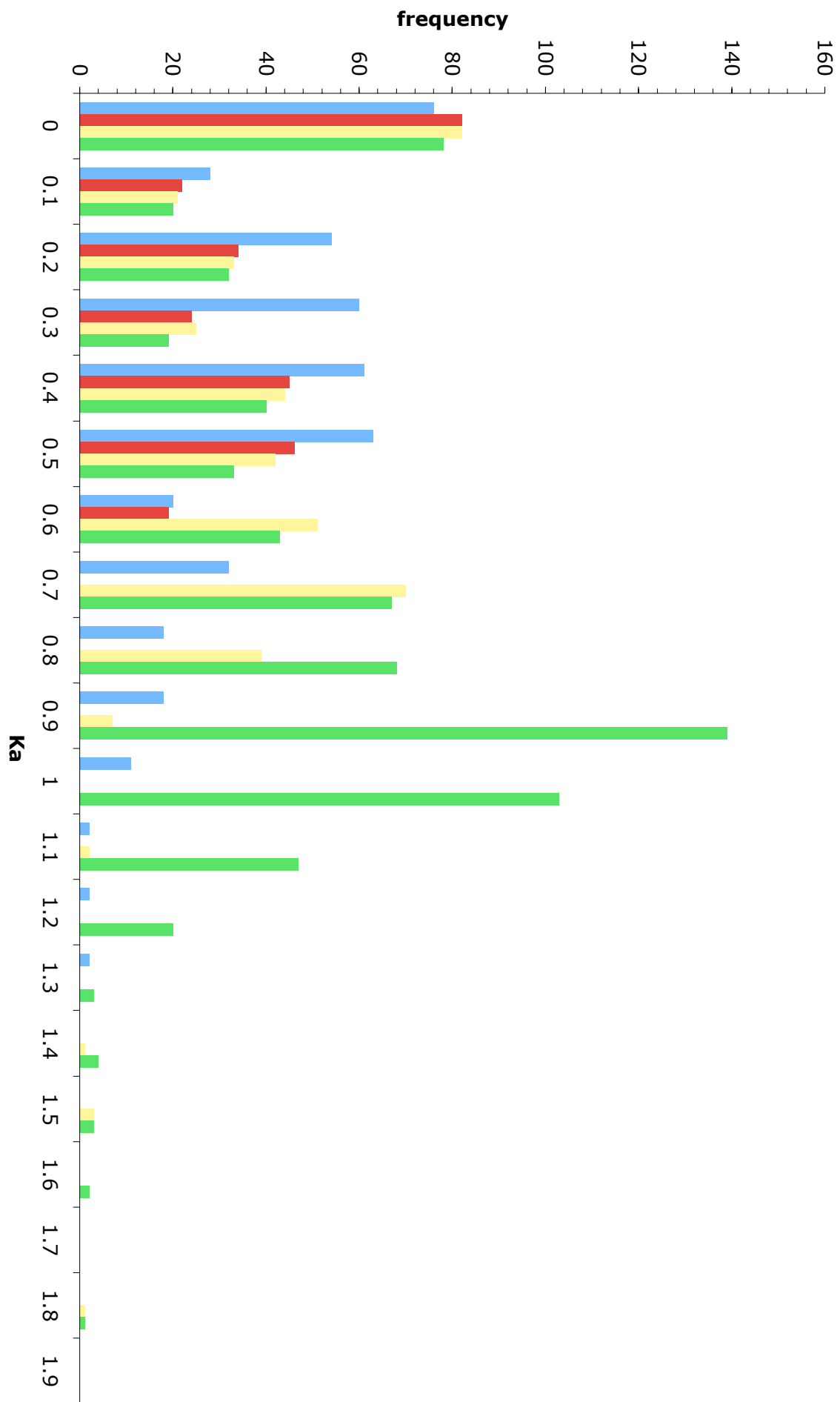

Supplement: Additional data file 3 — WGDs are illustrated in blue and SSDs are illustrated in red (found at 40% sequence identity), yellow (30% ID) and green (20% ID). [file gb-2007-8-10-r209-S3.pdf]
